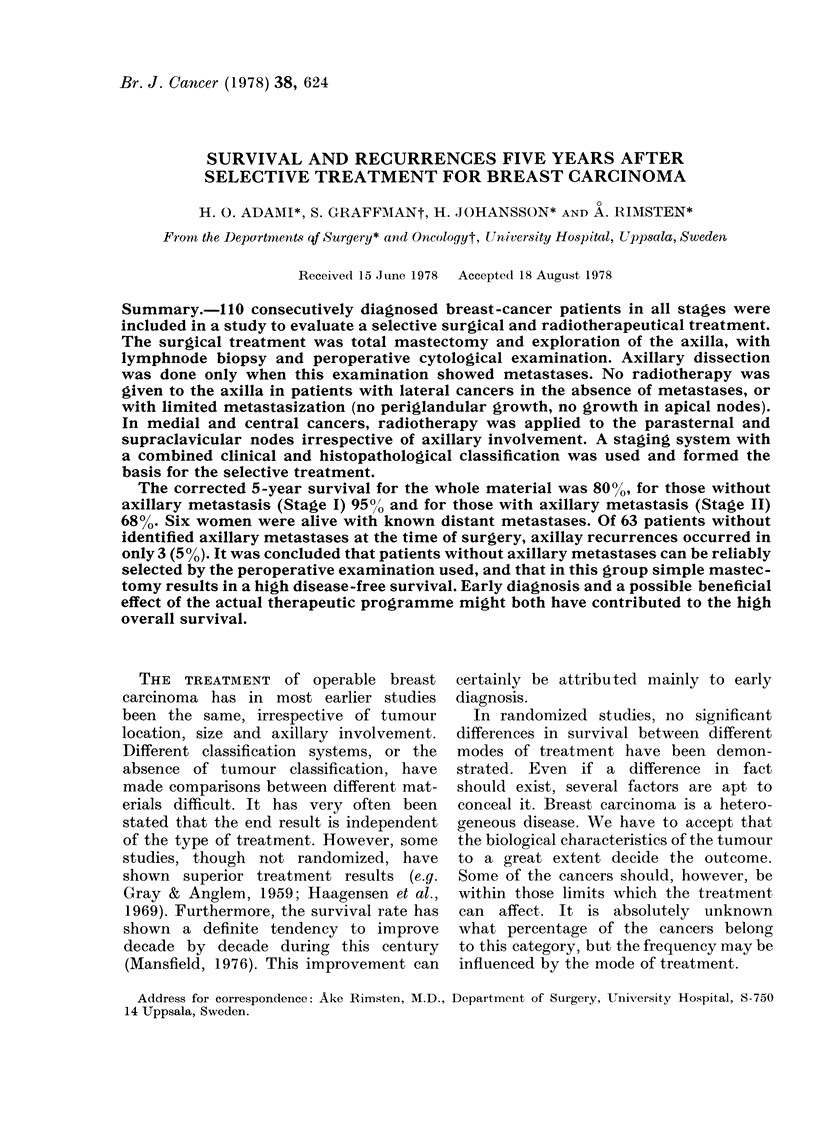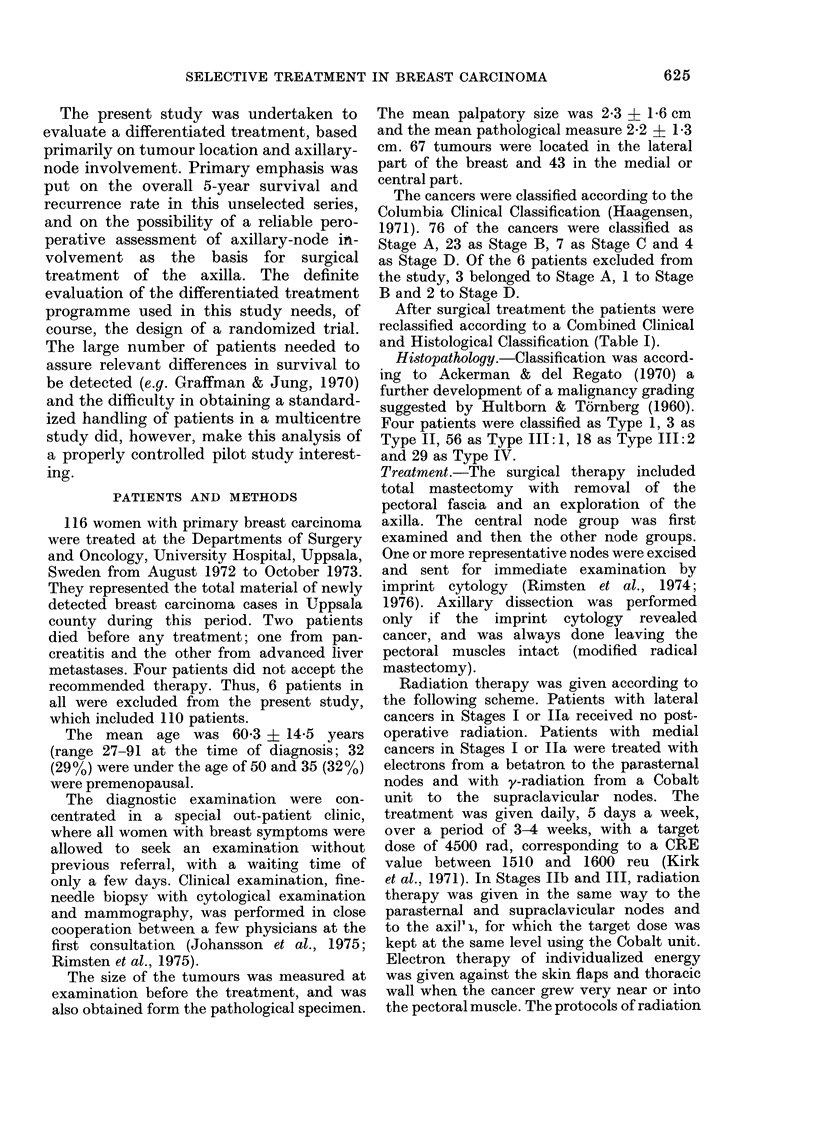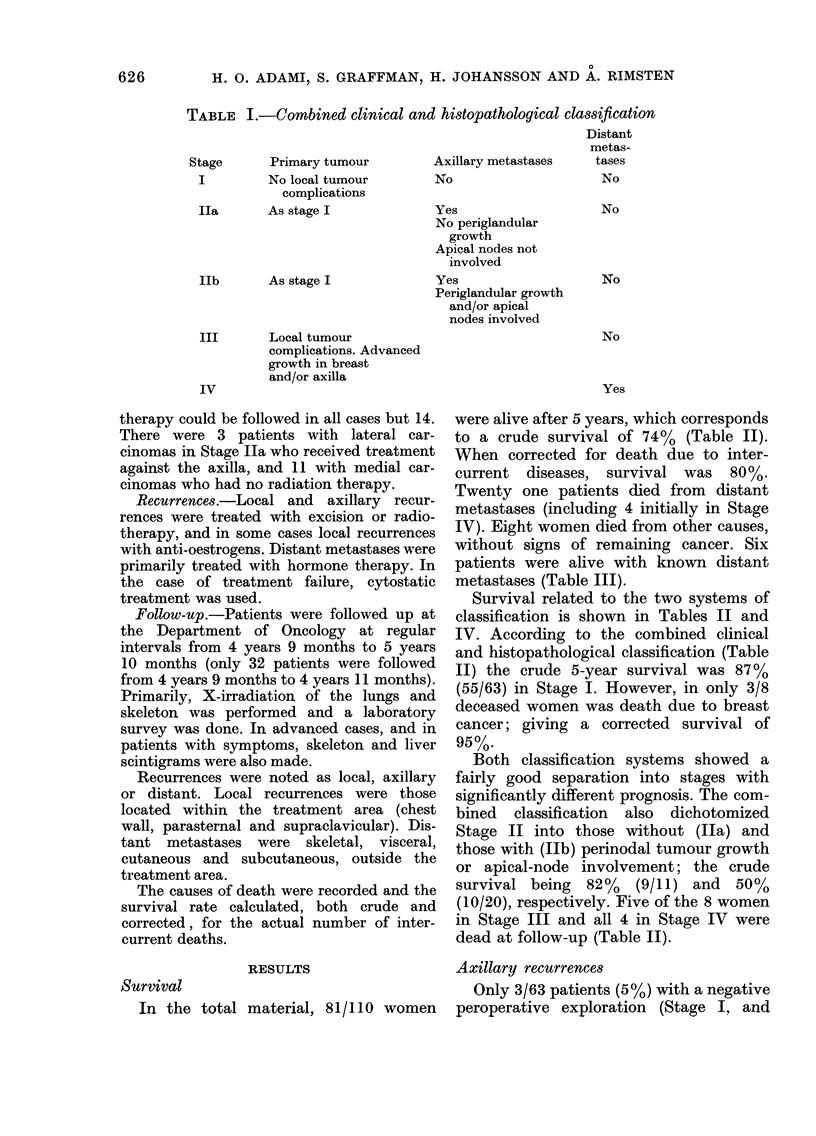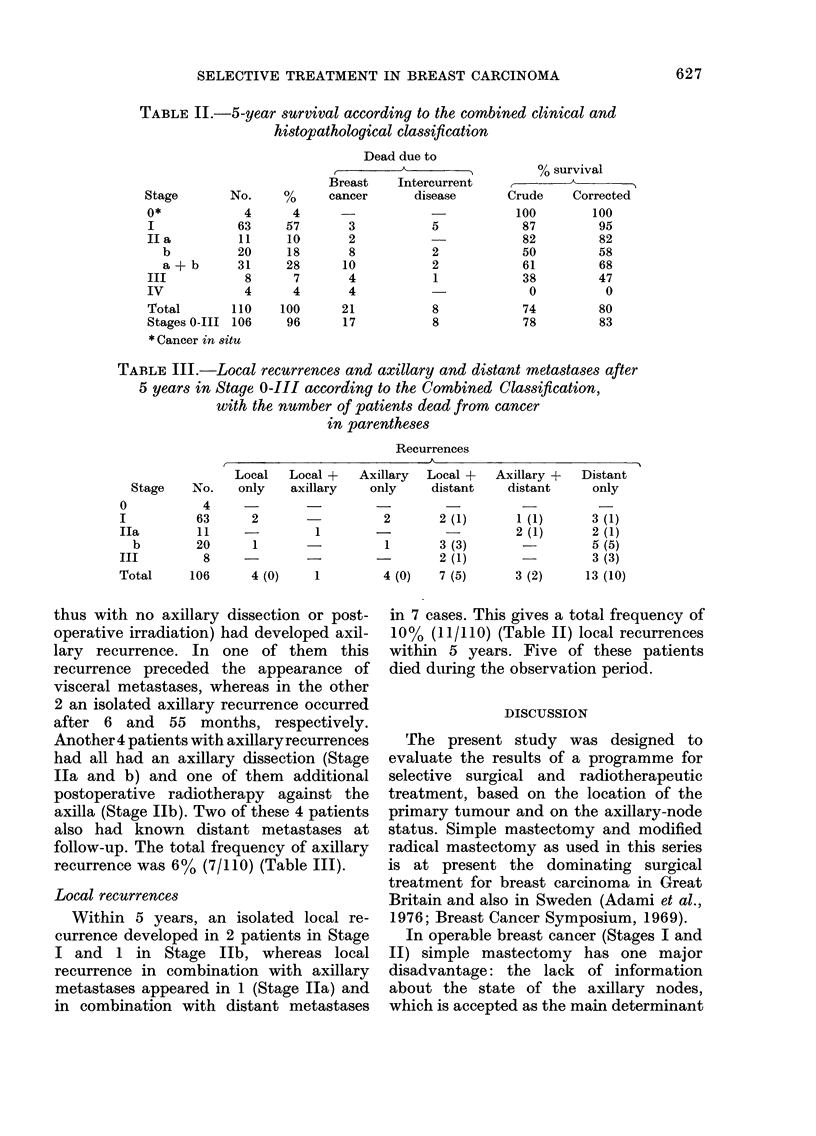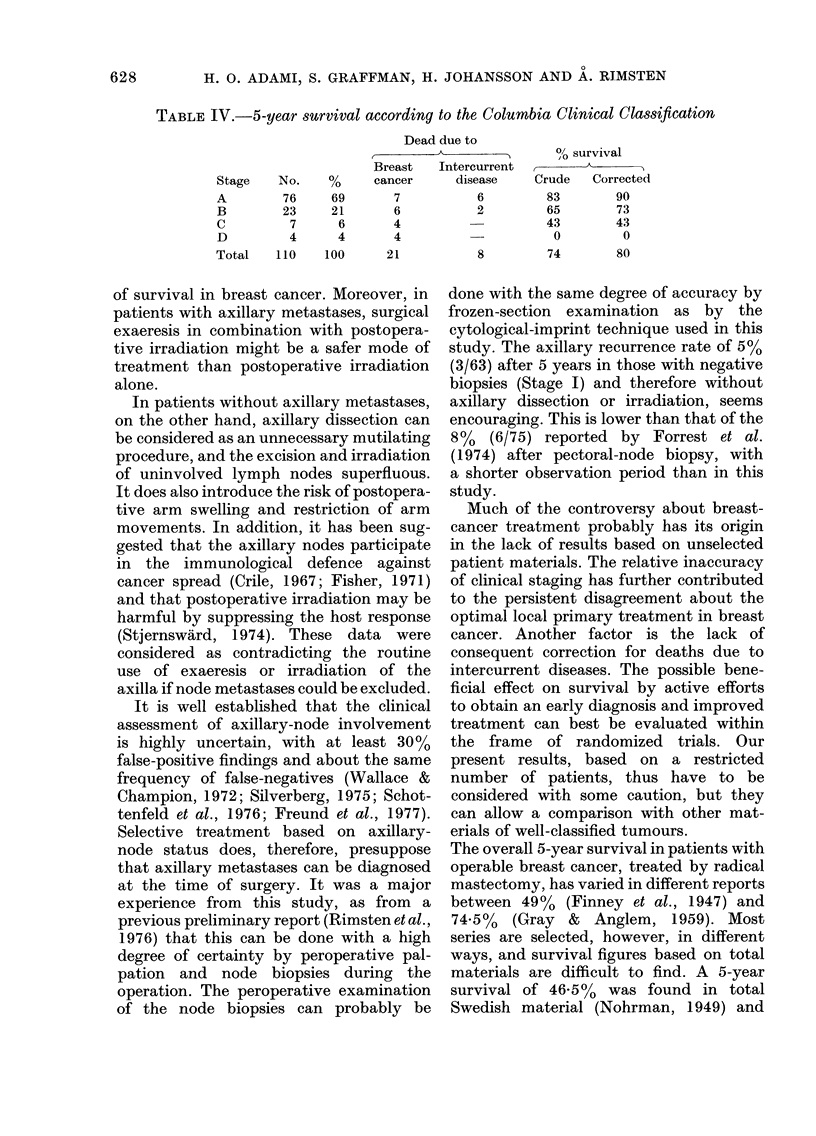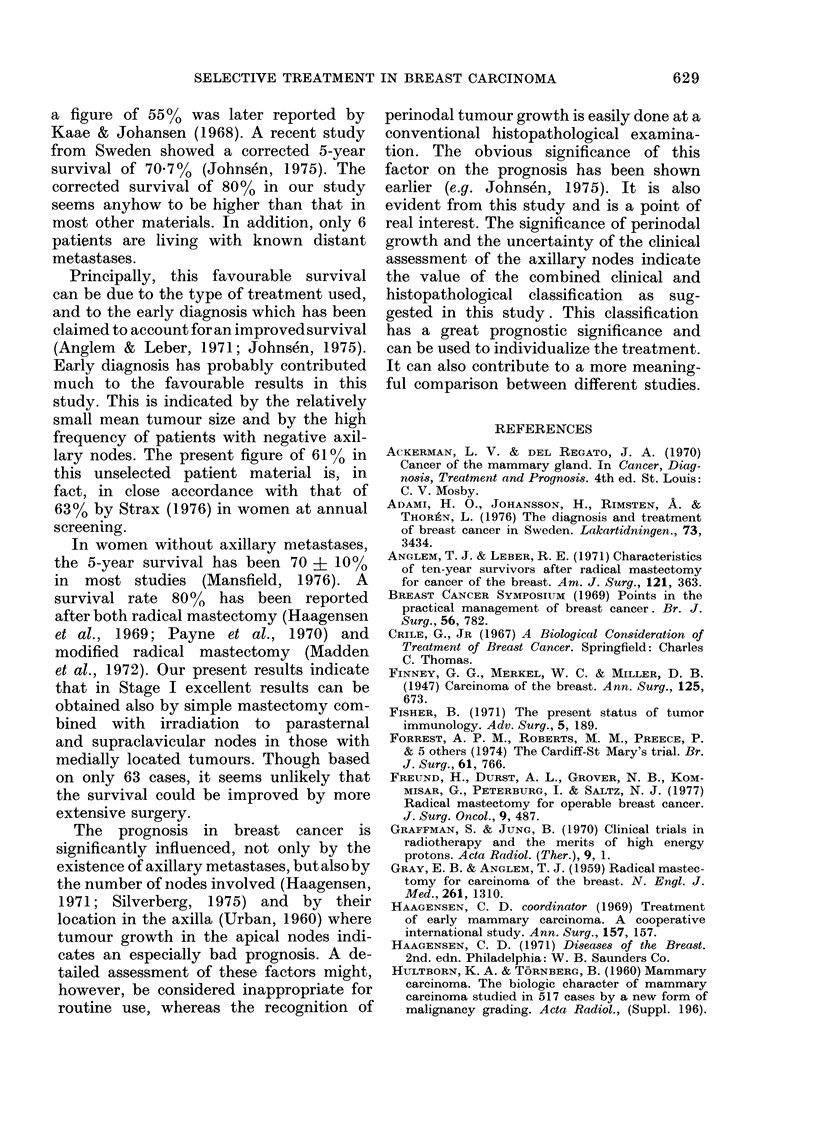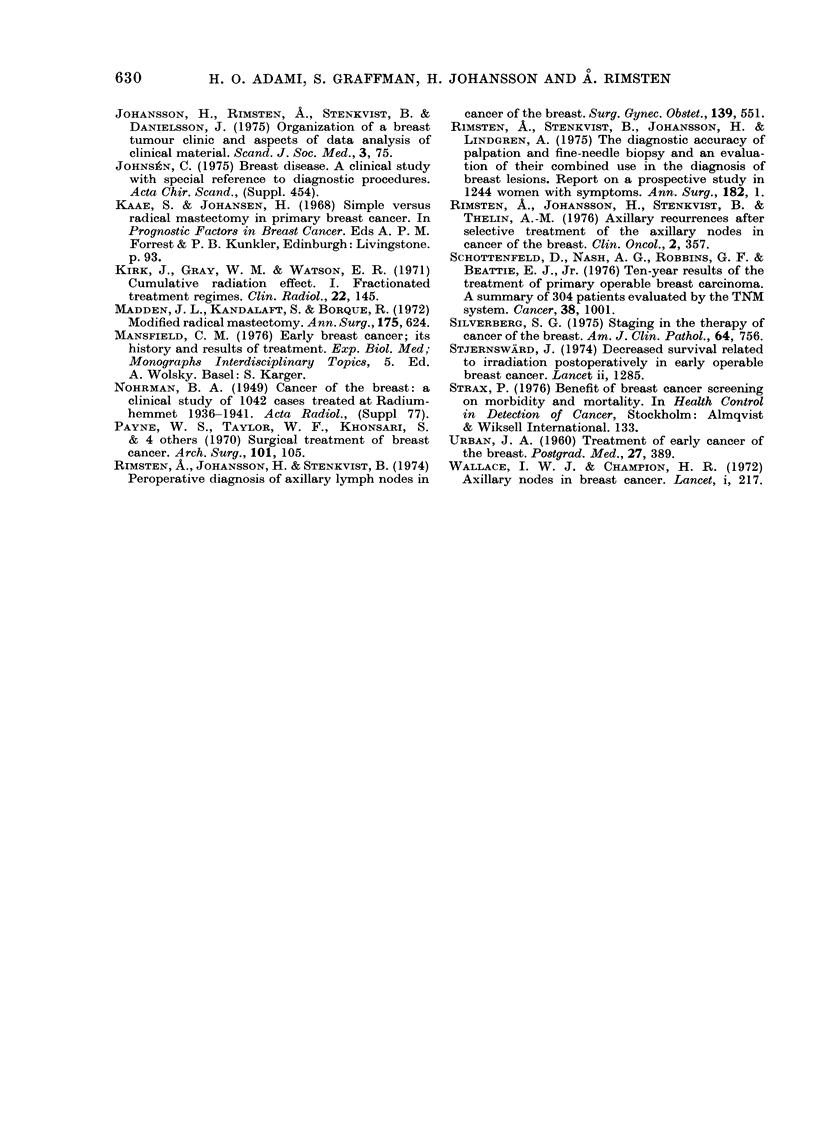# Survival and recurrences five years after selective treatment for breast carcinoma.

**DOI:** 10.1038/bjc.1978.259

**Published:** 1978-11

**Authors:** H. O. Adami, S. Graffman, H. Johansson, A. Rimsten

## Abstract

110 consecutively diagnosed breast-cancer patients in all stages were included in a study to evaluate a selective surgical and radiotherapeutical treatment. The surgical treatment was total mastectomy and exploration of the axilla, with lymphnode biopsy and peroperative cytological examination. Axillary dissection was done only when this examination showed metastases. No radiotherapy was given to the axilla in patients with lateral cancers in the absence of metastases, or with limited metastasization (no periglandular growth, no growth in apical nodes). In medial and central cancers, radiotherapy was applied to the parasternal and supraclavicular nodes irrespective of axillary involvement. A staging system with a combined clinical and histopathological classification was used and formed the basis for the selective treatment. The corrected 5-year survival for the whole material was 80%, for those without axillary metastasis (Stage I) 95% and for those with axillary metastasis (Stage II) 68%. Six women were alive with known distant metastases. Of 63 patients without identified axillary metastases at the time of surgery, axillary recurrences occurred in only 3 (5%). It was concluded that patients without axillary metastases can be reliably selected by the peroperative examination used, and that in this group simple mastectomy results in a high disease-free survival. Early diagnosis and a possible beneficial effect of the actual therapeutic programme might both have contributed to the high overall survival.


*Br. J. Cancer* (1978) **38**, 624

### SURVIVAL AND RECURRENCES FIVE YEARS AFTER SELECTIVE TREATMENT FOR BREAST CARCINOMA

H. O. ADAMI*, S. GRAFFMAN†, H. JOHANSSON* AND Å. RIMSTEN*

*From the Departments of Surgery* and Oncology†, University Hospital, Uppsala, Sweden*

Received 15 June 1978 Accepted 18 August 1978

**Summary.—110 consecutively diagnosed breast-cancer patients in all stages were included in a study to evaluate a selective surgical and radiotherapeutical treatment. The surgical treatment was total mastectomy and exploration of the axilla, with lymphnode biopsy and peroperative cytological examination. Axillary dissection was done only when this examination showed metastases. No radiotherapy was given to the axilla in patients with lateral cancers in the absence of metastases, or with limited metastasization (no periglandular growth, no growth in apical nodes). In medial and central cancers, radiotherapy was applied to the parasternal and supraclavicular nodes irrespective of axillary involvement. A staging system with a combined clinical and histopathological classification was used and formed the basis for the selective treatment.**

**The corrected 5-year survival for the whole material was 80%, for those without axillary metastasis (Stage I) 95% and for those with axillary metastasis (Stage II) 68%. Six women were alive with known distant metastases. Of 63 patients without identified axillary metastases at the time of surgery, axillay recurrences occurred in only 3 (5%). It was concluded that patients without axillary metastases can be reliably selected by the peroperative examination used, and that in this group simple mastectomy results in a high disease-free survival. Early diagnosis and a possible beneficial effect of the actual therapeutic programme might both have contributed to the high overall survival.**

THE TREATMENT of operable breast carcinoma has in most earlier studies been the same, irrespective of tumour location, size and axillary involvement. Different classification systems, or the absence of tumour classification, have made comparisons between different materials difficult. It has very often been stated that the end result is independent of the type of treatment. However, some studies, though not randomized, have shown superior treatment results (*e.g.* Gray & Anglem, 1959; Haagensen *et al.*, 1969). Furthermore, the survival rate has shown a definite tendency to improve decade by decade during this century (Mansfield, 1976). This improvement can certainly be attributed mainly to early diagnosis.

In randomized studies, no significant differences in survival between different modes of treatment have been demonstrated. Even if a difference in fact should exist, several factors are apt to conceal it. Breast carcinoma is a heterogeneous disease. We have to accept that the biological characteristics of the tumour to a great extent decide the outcome. Some of the cancers should, however, be within those limits which the treatment can affect. It is absolutely unknown what percentage of the cancers belong to this category, but the frequency may be influenced by the mode of treatment.

Address for correspondence: Åke Rimsten, M.D., Department of Surgery, University Hospital, S-750 14 Uppsala, Sweden.

The present study was undertaken to evaluate a differentiated treatment, based primarily on tumour location and axillary-node involvement. Primary emphasis was put on the overall 5-year survival and recurrence rate in this unselected series, and on the possibility of a reliable pero-perative assessment of axillary-node involvement as the basis for surgical treatment of the axilla. The definite evaluation of the differentiated treatment programme used in this study needs, of course, the design of a randomized trial. The large number of patients needed to assure relevant differences in survival to be detected (*e.g.* Graffman & Jung, 1970) and the difficulty in obtaining a standardized handling of patients in a multicentre study did, however, make this analysis of a properly controlled pilot study interesting.

### PATIENTS AND METHODS

116 women with primary breast carcinoma were treated at the Departments of Surgery and Oncology, University Hospital, Uppsala, Sweden from August 1972 to October 1973. They represented the total material of newly detected breast carcinoma cases in Uppsala county during this period. Two patients died before any treatment; one from pancreatitis and the other from advanced liver metastases. Four patients did not accept the recommended therapy. Thus, 6 patients in all were excluded from the present study, which included 110 patients.

The mean age was 60·3 ± 14·5 years (range 27–91 at the time of diagnosis; 32 (29%) were under the age of 50 and 35 (32%) were premenopausal.

The diagnostic examination were concentrated in a special out-patient clinic, where all women with breast symptoms were allowed to seek an examination without previous referral, with a waiting time of only a few days. Clinical examination, fine-needle biopsy with cytological examination and mammography, was performed in close cooperation between a few physicians at the first consultation (Johansson *et al.*, 1975; Rimsten *et al.*, 1975).

The size of the tumours was measured at examination before the treatment, and was also obtained form the pathological specimen. The mean palpatory size was 2·3 ± 1·6 cm and the mean pathological measure 2·2 ± 1·3 cm. 67 tumours were located in the lateral part of the breast and 43 in the medial or central part.

The cancers were classified according to the Columbia Clinical Classification (Haagensen, 1971). 76 of the cancers were classified as Stage A, 23 as Stage B, 7 as Stage C and 4 as Stage D. Of the 6 patients excluded from the study, 3 belonged to Stage A, 1 to Stage B and 2 to Stage D.

After surgical treatment the patients were reclassified according to a Combined Clinical and Histological Classification (Table I).

*Histopathology.*—Classification was according to Ackerman & del Regato (1970) a further development of a malignancy grading suggested by Hultborn & Törnberg (1960). Four patients were classified as Type 1, 3 as Type II, 56 as Type III:1, 18 as Type III:2 and 29 as Type IV.

*Treatment.*—The surgical therapy included total mastectomy with removal of the pectoral fascia and an exploration of the axilla. The central node group was first examined and then the other node groups. One or more representative nodes were excised and sent for immediate examination by imprint cytology (Rimsten *et al.*, 1974; 1976). Axillary dissection was performed only if the imprint cytology revealed cancer, and was always done leaving the pectoral muscles intact (modified radical mastectomy).

Radiation therapy was given according to the following scheme. Patients with lateral cancers in Stages I or IIa received no post-operative radiation. Patients with medial cancers in Stages I or IIa were treated with electrons from a betatron to the parasternal nodes and with $\gamma$-radiation from a Cobalt unit to the supraclavicular nodes. The treatment was given daily, 5 days a week, over a period of 3–4 weeks, with a target dose of 4500 rad, corresponding to a CRE value between 1510 and 1600 reu (Kirk *et al.*, 1971). In Stages IIb and III, radiation therapy was given in the same way to the parasternal and supraclavicular nodes and to the axil'ı, for which the target dose was kept at the same level using the Cobalt unit. Electron therapy of individualized energy was given against the skin flaps and thoracic wall when the cancer grew very near or into the pectoral muscle. The protocols of radiation

TABLE I.—*Combined clinical and histopathological classification*

| Stage | Primary tumour | Axillary metastases | Distant metastases |
|---|---|---|---|
| I | No local tumour complications | No | No |
| IIa | As stage I | Yes<br>No periglandular growth<br>Apical nodes not involved | No |
| IIb | As stage I | Yes<br>Periglandular growth and/or apical nodes involved | No |
| III | Local tumour complications. Advanced growth in breast and/or axilla | | No |
| IV | | | Yes |

therapy could be followed in all cases but 14. There were 3 patients with lateral carcinomas in Stage IIa who received treatment against the axilla, and 11 with medial carcinomas who had no radiation therapy.

*Recurrences.*—Local and axillary recurrences were treated with excision or radiotherapy, and in some cases local recurrences with anti-oestrogens. Distant metastases were primarily treated with hormone therapy. In the case of treatment failure, cytostatic treatment was used.

*Follow-up.*—Patients were followed up at the Department of Oncology at regular intervals from 4 years 9 months to 5 years 10 months (only 32 patients were followed from 4 years 9 months to 4 years 11 months). Primarily, X-irradiation of the lungs and skeleton was performed and a laboratory survey was done. In advanced cases, and in patients with symptoms, skeleton and liver scintigrams were also made.

Recurrences were noted as local, axillary or distant. Local recurrences were those located within the treatment area (chest wall, parasternal and supraclavicular). Distant metastases were skeletal, visceral, cutaneous and subcutaneous, outside the treatment area.

The causes of death were recorded and the survival rate calculated, both crude and corrected, for the actual number of intercurrent deaths.

### RESULTS

### *Survival*

In the total material, 81/110 women were alive after 5 years, which corresponds to a crude survival of 74% (Table II). When corrected for death due to intercurrent diseases, survival was 80%. Twenty one patients died from distant metastases (including 4 initially in Stage IV). Eight women died from other causes, without signs of remaining cancer. Six patients were alive with known distant metastases (Table III).

Survival related to the two systems of classification is shown in Tables II and IV. According to the combined clinical and histopathological classification (Table II) the crude 5-year survival was 87% (55/63) in Stage I. However, in only 3/8 deceased women was death due to breast cancer; giving a corrected survival of 95%.

Both classification systems showed a fairly good separation into stages with significantly different prognosis. The combined classification also dichotomized Stage II into those without (IIa) and those with (IIb) perinodal tumour growth or apical-node involvement; the crude survival being 82% (9/11) and 50% (10/20), respectively. Five of the 8 women in Stage III and all 4 in Stage IV were dead at follow-up (Table II).

### *Axillary recurrences*

Only 3/63 patients (5%) with a negative peroperative exploration (Stage I, and

TABLE II.—*5-year survival according to the combined clinical and histopathological classification*

| Stage | No. | % | Dead due to: Breast cancer | Dead due to: Intercurrent disease | % survival: Crude | % survival: Corrected |
|---|---|---|---|---|---|---|
| 0* | 4 | 4 | — | — | 100 | 100 |
| I | 63 | 57 | 3 | 5 | 87 | 95 |
| II a | 11 | 10 | 2 | — | 82 | 82 |
| b | 20 | 18 | 8 | 2 | 50 | 58 |
| a + b | 31 | 28 | 10 | 2 | 61 | 68 |
| III | 8 | 7 | 4 | 1 | 38 | 47 |
| IV | 4 | 4 | 4 | — | 0 | 0 |
| Total | 110 | 100 | 21 | 8 | 74 | 80 |
| Stages 0-III | 106 | 96 | 17 | 8 | 78 | 83 |

* Cancer *in situ*

TABLE III.—*Local recurrences and axillary and distant metastases after 5 years in Stage 0-III according to the Combined Classification, with the number of patients dead from cancer in parentheses*

| Stage | No. | Recurrences: Local only | Local + axillary | Axillary only | Local + distant | Axillary + distant | Distant only |
|---|---|---|---|---|---|---|---|
| 0 | 4 | — | — | — | — | — | — |
| I | 63 | 2 | — | 2 | 2 (1) | 1 (1) | 3 (1) |
| IIa | 11 | — | 1 | — | — | 2 (1) | 2 (1) |
| b | 20 | 1 | — | 1 | 3 (3) | — | 5 (5) |
| III | 8 | — | — | — | 2 (1) | — | 3 (3) |
| Total | 106 | 4 (0) | 1 | 4 (0) | 7 (5) | 3 (2) | 13 (10) |

thus with no axillary dissection or post-operative irradiation) had developed axillary recurrence. In one of them this recurrence preceded the appearance of visceral metastases, whereas in the other 2 an isolated axillary recurrence occurred after 6 and 55 months, respectively. Another 4 patients with axillary recurrences had all had an axillary dissection (Stage IIa and b) and one of them additional postoperative radiotherapy against the axilla (Stage IIb). Two of these 4 patients also had known distant metastases at follow-up. The total frequency of axillary recurrence was 6% (7/110) (Table III).

*Local recurrences*

Within 5 years, an isolated local recurrence developed in 2 patients in Stage I and 1 in Stage IIb, whereas local recurrence in combination with axillary metastases appeared in 1 (Stage IIa) and in combination with distant metastases in 7 cases. This gives a total frequency of 10% (11/110) (Table II) local recurrences within 5 years. Five of these patients died during the observation period.

### DISCUSSION

The present study was designed to evaluate the results of a programme for selective surgical and radiotherapeutic treatment, based on the location of the primary tumour and on the axillary-node status. Simple mastectomy and modified radical mastectomy as used in this series is at present the dominating surgical treatment for breast carcinoma in Great Britain and also in Sweden (Adami *et al.*, 1976; Breast Cancer Symposium, 1969).

In operable breast cancer (Stages I and II) simple mastectomy has one major disadvantage: the lack of information about the state of the axillary nodes, which is accepted as the main determinant

TABLE IV.—*5-year survival according to the Columbia Clinical Classification*

| | | | Dead due to | | % survival | |
|---|---|---|---|---|---|---|
| Stage | No. | % | Breast cancer | Intercurrent disease | Crude | Corrected |
| A | 76 | 69 | 7 | 6 | 83 | 90 |
| B | 23 | 21 | 6 | 2 | 65 | 73 |
| C | 7 | 6 | 4 | — | 43 | 43 |
| D | 4 | 4 | 4 | — | 0 | 0 |
| Total | 110 | 100 | 21 | 8 | 74 | 80 |

of survival in breast cancer. Moreover, in patients with axillary metastases, surgical exaeresis in combination with postoperative irradiation might be a safer mode of treatment than postoperative irradiation alone.

In patients without axillary metastases, on the other hand, axillary dissection can be considered as an unnecessary mutilating procedure, and the excision and irradiation of uninvolved lymph nodes superfluous. It does also introduce the risk of postoperative arm swelling and restriction of arm movements. In addition, it has been suggested that the axillary nodes participate in the immunological defence against cancer spread (Crile, 1967; Fisher, 1971) and that postoperative irradiation may be harmful by suppressing the host response (Stjernswärd, 1974). These data were considered as contradicting the routine use of exaeresis or irradiation of the axilla if node metastases could be excluded.

It is well established that the clinical assessment of axillary-node involvement is highly uncertain, with at least 30% false-positive findings and about the same frequency of false-negatives (Wallace & Champion, 1972; Silverberg, 1975; Schottenfeld *et al.*, 1976; Freund *et al.*, 1977). Selective treatment based on axillary-node status does, therefore, presuppose that axillary metastases can be diagnosed at the time of surgery. It was a major experience from this study, as from a previous preliminary report (Rimsten *et al.*, 1976) that this can be done with a high degree of certainty by peroperative palpation and node biopsies during the operation. The peroperative examination of the node biopsies can probably be done with the same degree of accuracy by frozen-section examination as by the cytological-imprint technique used in this study. The axillary recurrence rate of 5% (3/63) after 5 years in those with negative biopsies (Stage I) and therefore without axillary dissection or irradiation, seems encouraging. This is lower than that of the 8% (6/75) reported by Forrest *et al.* (1974) after pectoral-node biopsy, with a shorter observation period than in this study.

Much of the controversy about breast-cancer treatment probably has its origin in the lack of results based on unselected patient materials. The relative inaccuracy of clinical staging has further contributed to the persistent disagreement about the optimal local primary treatment in breast cancer. Another factor is the lack of consequent correction for deaths due to intercurrent diseases. The possible beneficial effect on survival by active efforts to obtain an early diagnosis and improved treatment can best be evaluated within the frame of randomized trials. Our present results, based on a restricted number of patients, thus have to be considered with some caution, but they can allow a comparison with other materials of well-classified tumours.

The overall 5-year survival in patients with operable breast cancer, treated by radical mastectomy, has varied in different reports between 49% (Finney *et al.*, 1947) and 74·5% (Gray & Anglem, 1959). Most series are selected, however, in different ways, and survival figures based on total materials are difficult to find. A 5-year survival of 46·5% was found in total Swedish material (Nohrman, 1949) and a figure of 55% was later reported by Kaae & Johansen (1968). A recent study from Sweden showed a corrected 5-year survival of 70·7% (Johnsén, 1975). The corrected survival of 80% in our study seems anyhow to be higher than that in most other materials. In addition, only 6 patients are living with known distant metastases.

Principally, this favourable survival can be due to the type of treatment used, and to the early diagnosis which has been claimed to account for an improved survival (Anglem & Leber, 1971; Johnsén, 1975). Early diagnosis has probably contributed much to the favourable results in this study. This is indicated by the relatively small mean tumour size and by the high frequency of patients with negative axillary nodes. The present figure of 61% in this unselected patient material is, in fact, in close accordance with that of 63% by Strax (1976) in women at annual screening.

In women without axillary metastases, the 5-year survival has been 70 ± 10% in most studies (Mansfield, 1976). A survival rate 80% has been reported after both radical mastectomy (Haagensen *et al.*, 1969; Payne *et al.*, 1970) and modified radical mastectomy (Madden *et al.*, 1972). Our present results indicate that in Stage I excellent results can be obtained also by simple mastectomy combined with irradiation to parasternal and supraclavicular nodes in those with medially located tumours. Though based on only 63 cases, it seems unlikely that the survival could be improved by more extensive surgery.

The prognosis in breast cancer is significantly influenced, not only by the existence of axillary metastases, but also by the number of nodes involved (Haagensen, 1971; Silverberg, 1975) and by their location in the axilla (Urban, 1960) where tumour growth in the apical nodes indicates an especially bad prognosis. A detailed assessment of these factors might, however, be considered inappropriate for routine use, whereas the recognition of perinodal tumour growth is easily done at a conventional histopathological examination. The obvious significance of this factor on the prognosis has been shown earlier (*e.g.* Johnsén, 1975). It is also evident from this study and is a point of real interest. The significance of perinodal growth and the uncertainty of the clinical assessment of the axillary nodes indicate the value of the combined clinical and histopathological classification as suggested in this study. This classification has a great prognostic significance and can be used to individualize the treatment. It can also contribute to a more meaningful comparison between different studies.

### REFERENCES

Ackerman, L. V. & del Regato, J. A. (1970) Cancer of the mammary gland. In *Cancer, Diagnosis, Treatment and Prognosis.* 4th ed. St. Louis: C. V. Mosby.

Adami, H. O., Johansson, H., Rimsten, Å. & Thorén, L. (1976) The diagnosis and treatment of breast cancer in Sweden. *Lakartidningen.*, **73**, 3434.

Anglem, T. J. & Leber, R. E. (1971) Characteristics of ten-year survivors after radical mastectomy for cancer of the breast. *Am. J. Surg.*, **121**, 363.

Breast Cancer Symposium (1969) Points in the practical management of breast cancer. *Br. J. Surg.*, **56**, 782.

Crile, G., Jr (1967) *A Biological Consideration of Treatment of Breast Cancer.* Springfield: Charles C. Thomas.

Finney, G. G., Merkel, W. C. & Miller, D. B. (1947) Carcinoma of the breast. *Ann. Surg.*, **125**, 673.

Fisher, B. (1971) The present status of tumor immunology. *Adv. Surg.*, **5**, 189.

Forrest, A. P. M., Roberts, M. M., Preece, P. & 5 others (1974) The Cardiff-St Mary's trial. *Br. J. Surg.*, **61**, 766.

Freund, H., Durst, A. L., Grover, N. B., Kommisar, G., Peterburg, I. & Saltz, N. J. (1977) Radical mastectomy for operable breast cancer. *J. Surg. Oncol.*, **9**, 487.

Graffman, S. & Jung, B. (1970) Clinical trials in radiotherapy and the merits of high energy protons. *Acta Radiol.* (*Ther.*), **9**, 1.

Gray, E. B. & Anglem, T. J. (1959) Radical mastectomy for carcinoma of the breast. *N. Engl. J. Med.*, **261**, 1310.

Haagensen, C. D. *coordinator* (1969) Treatment of early mammary carcinoma. A cooperative international study. *Ann. Surg.*, **157**, 157.

Haagensen, C. D. (1971) *Diseases of the Breast.* 2nd. edn. Philadelphia: W. B. Saunders Co.

Hultborn, K. A. & Törnberg, B. (1960) Mammary carcinoma. The biologic character of mammary carcinoma studied in 517 cases by a new form of malignancy grading. *Acta Radiol.*, (Suppl. 196).

JOHANSSON, H., RIMSTEN, Å., STENKVIST, B. & DANIELSSON, J. (1975) Organization of a breast tumour clinic and aspects of data analysis of clinical material. *Scand. J. Soc. Med.*, **3**, 75.

JOHNSÉN, C. (1975) Breast disease. A clinical study with special reference to diagnostic procedures. *Acta Chir. Scand.*, (Suppl. 454).

KAAE, S. & JOHANSEN, H. (1968) Simple versus radical mastectomy in primary breast cancer. In *Prognostic Factors in Breast Cancer*. Eds A. P. M. Forrest & P. B. Kunkler, Edinburgh: Livingstone. p. 93.

KIRK, J., GRAY, W. M. & WATSON, E. R. (1971) Cumulative radiation effect. I. Fractionated treatment regimes. *Clin. Radiol.*, **22**, 145.

MADDEN, J. L., KANDALAFT, S. & BORQUE, R. (1972) Modified radical mastectomy. *Ann. Surg.*, **175**, 624.

MANSFIELD, C. M. (1976) Early breast cancer; its history and results of treatment. *Exp. Biol. Med; Monographs Interdisciplinary Topics*, 5. Ed. A. Wolsky. Basel: S. Karger.

NOHRMAN, B. A. (1949) Cancer of the breast: a clinical study of 1042 cases treated at Radiumhemmet 1936–1941. *Acta Radiol.*, (Suppl 77).

PAYNE, W. S., TAYLOR, W. F., KHONSARI, S. & 4 others (1970) Surgical treatment of breast cancer. *Arch. Surg.*, **101**, 105.

RIMSTEN, Å., JOHANSSON, H. & STENKVIST, B. (1974) Peroperative diagnosis of axillary lymph nodes in cancer of the breast. *Surg. Gynec. Obstet.*, **139**, 551.

RIMSTEN, Å., STENKVIST, B., JOHANSSON, H. & LINDGREN, A. (1975) The diagnostic accuracy of palpation and fine-needle biopsy and an evaluation of their combined use in the diagnosis of breast lesions. Report on a prospective study in 1244 women with symptoms. *Ann. Surg.*, **182**, 1.

RIMSTEN, Å., JOHANSSON, H., STENKVIST, B. & THELIN, A.-M. (1976) Axillary recurrences after selective treatment of the axillary nodes in cancer of the breast. *Clin. Oncol.*, **2**, 357.

SCHOTTENFELD, D., NASH, A. G., ROBBINS, G. F. & BEATTIE, E. J., Jr. (1976) Ten-year results of the treatment of primary operable breast carcinoma. A summary of 304 patients evaluated by the TNM system. *Cancer*, **38**, 1001.

SILVERBERG, S. G. (1975) Staging in the therapy of cancer of the breast. *Am. J. Clin. Pathol.*, **64**, 756.

STJERNSWÄRD, J. (1974) Decreased survival related to irradiation postoperatively in early operable breast cancer. *Lancet* ii, 1285.

STRAX, P. (1976) Benefit of breast cancer screening on morbidity and mortality. In *Health Control in Detection of Cancer*, Stockholm: Almqvist & Wiksell International. 133.

URBAN, J. A. (1960) Treatment of early cancer of the breast. *Postgrad. Med.*, **27**, 389.

WALLACE, I. W. J. & CHAMPION, H. R. (1972) Axillary nodes in breast cancer. *Lancet*, i, 217.